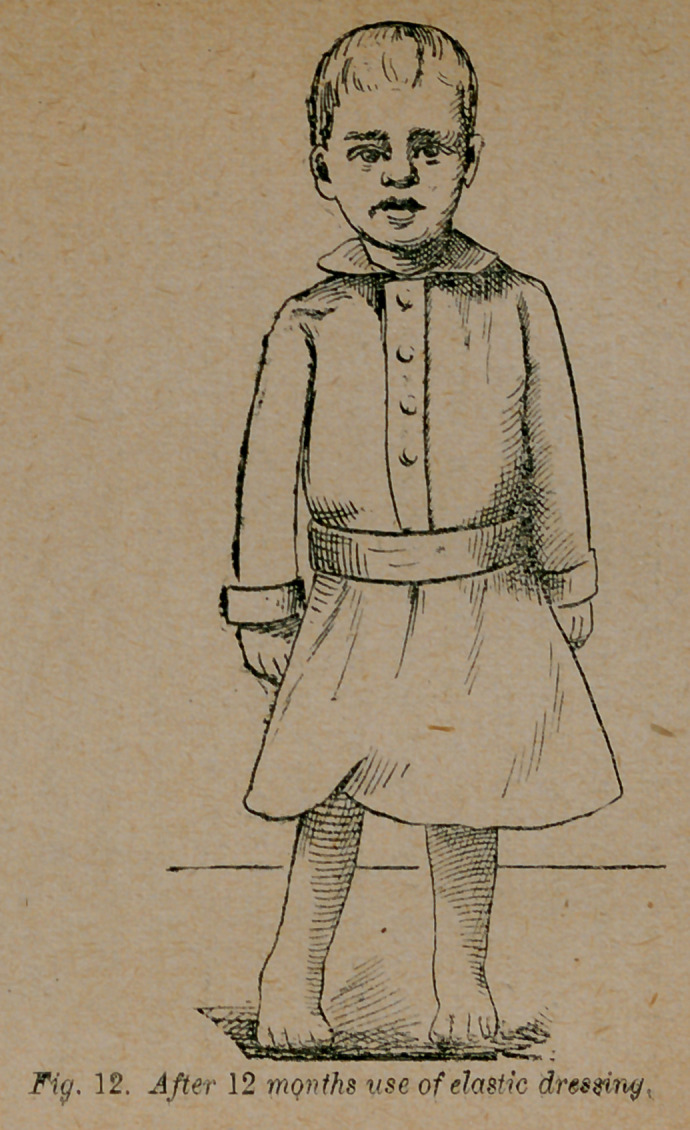# Treatment of Talipes Equino-Varus

**Published:** 1893-01

**Authors:** C. W. Barrier

**Affiliations:** Columbus, Ga.


ATLANTA
MEDICAL AND SURGICAL JOURNAL.

VOL. IX. JANUARY, 1893. No. 11.

EDITED BY
LUTHER B. GRANDY, M. D., AND MILLER B. HUTCHINS, M. D.,
WITH THE CO-OPERATION OF
H. V. M. MILLER, M. D., LL. D., VIRGIL O. HARDON, M. D., AND
FLOYD W. McRAE, M. D.

### ORIGINAL COMMUNICATIONS.

### TREATMENT OF TALIPES EQUINO-VARUS.*

BY C. W. BARRIER, M. D.,
COLUMBUS, GA.

GENTLEMEN—This is Master Joe Hutchinson, of Johnson county, Georgia, whose condition is interesting, as representing the bad effect of the use of the fixed dressings, such as plaster Paris and steel braces. He was at birth a double talipes equino-varus His father being deformed in the same way, and having experienced the disadvantage of being a cripple his whole life, placed this his first son in the care of an institution, which offers various advantages for the care of such deformities. At the early age of three months steel braces were applied, preventing all muscular exercise of the limbs, and pulling the toe *down* and out till the case was converted into one of extreme equinus. Then braces were applied to pull the toe up. During this time he

*Read before the Tri-State Medical Society of Georgia, Alabama and Tennessee, Chattanooga, Tenn., Oct. 25, 1892.

made three visits to the institution. At the end of three years of this treatment, the deformity still not being corrected, the father made and applied such braces as you now see on him. He has continued to wear these, until he is now sixteen years old. He has never left them off, and he is not now able to make a step without them. Thus, for nearly half an average lifetime, this patient has been braced and strapped and both limbs cramped in such a manner that exercise, with its consequent development has been impossible. Is it any wonder that he must still crawl like a babe when the braces are off ? See how atrophied his limbs are. See how he walks, using himself as if he were on peg legs.

In my estimation, the error in the treatment of this case is twofold ; First, tenotomy should have been performed, and the deformity removed in at least twelve months ; and the child's limbs left uncramped, so that muscular exercise and development might be possible. Second, *this brace should never have been applied in such a way as to prevent the full exercise of these weak muscles so long ; nor should it have pulled the toe down in such a way as to increase the equinus; since the equinus and varus may have been corrected at the same time, by a single dressing, so simple as is shown in figure* 11, *which promotes the exercise of these muscles.*

If this had been an isolated case, I would not have brought him before you, but I have met many such.

CASE 2.—Miss O. J. of Long Pond, Ga., a congenital talipes equino-varus in one foot was at the age of two years placed under the care of the same institution. A brace was applied with a steel rod projecting below the heel, making it impossible for the foot even to rest on the floor. In this way the patient was prevented from standing or walking for three months, making exercise, with its consequent development, impossible. No benefit was derived from the weight of the body in pressing the foot straight, while the case was converted into one of equinus. The child made a second visit to the institution. Tenotomy was performed and different braces were applied to reduce the equinus. After five years of such treatment the patient was still uncured and the braces were discontinued. Last January, when she was thirteen years old, assisted by Dr. Henry McArthur of Long Pond, I operated on her. We used cocaine and the patient said it gave her little or no pain, as many have testified. We used no force nor violence to wrench the foot into shape, but simply applied an elastic

CASE III.

*Fig.* 1. *After treated with braces.* *Fig.* 2. *After a few months of the elastic treatment but not completed.*

strap. In fifteen days, she could walk flat-footed, and we dismissed the case. She continues to go flat-footed with only a common shoe. The limb has begun to develop under the influence of muscular contraction, and will be a well shaped and useful leg.

CASE 3.—Master J. P., of Dodge county, Ga., was placed under the care of the same institution when only a few months old.

The same process of steel bracing was employed. After months of suffering, the parents being discouraged, dismissed the treatment. On the 4th of June, 1891, he appeared as in figure 1. At this time, with the assistance of Dr. J. D. Henman of Eastman, Ga., I operated upon him. I made frequent attempts to use fixed dressing, but all to no avail. The foot would turn over in them in spite of all that could be done, and the shoes would turn over. In no way could I force the sole of the foot to come down flat on the ground until I made and applied a simple elastic dressing. In ten days he could stand on his feet and walk some. In this way the weight of the body was employed as an auxiliary in pressing the feet straight, and when he walked muscular exercise began to arrest the process of atrophy, and develop the limb. Now after seventeen months treatment, he is able to stand as shown in figure 2, and is beginning to walk with no dressing on his feet. One foot is completely cured and by next spring the other will be cured so he can go without shoes.

Regular surgeons of ability seem never to have profited by the experience of Dr. Sayre, and seem to pay no attention to his warning against the use of fixed dressings; such as plaster Paris and steel braces. But they often remark that the plaster Paris dressing is the ideal one. Others consult advertisements for advice as to the best braces and order the one which is recommended by the most extravagant advertisement. These braces occupy about the same relation to legitimate orthopedic surgery as patent medicines do to the honorable practice of our profession.

CASE 4.—T. F. of Phenix City, Ala., originally a congenital talipes equino-varus in both feet, had tenotomy performed two or three times in each foot while a child. This was done by a highly educated surgeon, for whose ability I have the utmost regard. He ordered and applied these excellently (?) complicated braces; and the patient continued the use of different kinds of braces until he was twenty years and eight months of age when he applied to me for treatment. The deformity had been corrected in one foot, but the other was as represented in fig. 3. He had been using braces on this foot thirteen years, and at the time he applied for treatment, he could not go without them. The braces had caused such corns and ulcers that he was unable to work more than three or four days each week. Œdema of the foot and leg had set in, making the condition of the patient pitiable indeed. In the face of this history of failure, and advanced age, I operated on him and threw away the brace, ap-

*CASE IV.*

*Fig. 3. After four or five operations and thirteen years use of brace.*

*Fig. 4. After five months of the elastic treatment.*

plying a simple form of the elastic dressing. With cocaine the tenotomy was so painless, and the patient seeing at each operation the increased improvement in the condition of the foot, insisted on my repeating the operation every three or four days. This I did several times, making extensive subcutaneous incisions all through the inner side of the foot. By this procedure and the constant traction of the elastic, in four months he was able to leave off all dressing and return to his work-bench. He wears only a simple shoe, and his feet give him no trouble.

CASE 5.—A. W. of Phenix City, Ala., a case of double talipes equino-varus from birth, was treated with braces from

CASE V.

*Fig.* 5. *After ten years use of brace.*

*Fig.* 6. *After four months elastic treatment.*

the time he was six years old until he was sixteen. In January, 1889, he applied to me for treatment. He had been cured in one foot, but was badly deformed in the other as in fig. 5. This foot was so defective, that he had not walked the distance of a half mile at a time, and had never done a half day's work. In this foot the deformity was so high up in the ankle joint, that when he made an effort to walk, the whole side of the foot dragged through the mud. Assisted by Dr. W. W. Bloodworth of Opelika, Ala., I operated on him. After four months' use of the simplest dressings I dismissed the case cured, with a foot as straight as any one's foot, as the right foot in fig. 6. A few days afterward I remember seeing him playing ball. Five months after the operation, I saw him following the fireman's parade, at a

CASE VI.

*Fig. 7. After operation and several months use of the brace.* *Fig. 8. With elastic dressing.* *Fig. 9. After three months of the elastic treatment but not dismissed.*

distance of a mile and a half from his father's house. Since I dismissed the case he has never worn anything but a common shoe, and has made a good hand at a work in which he stands on his feet twelve hours each day.

CASE 6.—Miss R. Y., of Harrison, Ga., was a congenital talipes equino-varus in one foot as in fig. 7. At the age of two years an eminent surgeon performed tenotomy, and applied the most approved and extravagantly advertised brace that could be had. She wore these with a most heart-rending history of suffering and failure. When she was twelve years old, assisted by Drs. T. E. Vickery, W. F. and T. E. Peacock, I performed tenotomy. I applied a simple elastic dressing as shown in fig. 8. It consists of a stiff soled shoe (I no longer use a jointed soled shoe) with a small steel projection. An elastic strap is attached to this steel, which extends to the limb, and is fastened to it above the knee by a soft bandage of cotton flannel. This simple contrivance is so constructed that it pulls the foot *up* (not *down*, as was practiced in the case of this young man) and out at the same time, and holds it in almost exact position. She was able to stand and walk with the dressing on in four days. She has now worn it three months, and is beginning to stand as shown in fig. 9, and can walk some without any dressing on. In a few months no one would suspect that she was deformed.

If other cases were necessary to illustrate the ill effect of these braces my manual would furnish it. I have not reported such cases as may have recovered without treatment, but I have reported only the more difficult cases, such as had been treated for months and years by intelligent and capable parties using fixed dressings. I have treated many other cases by the elastic method and have been able to control all except a negro boy fourteen years old; the only negro I have operated on.

CASE 7.—I submit one more case representing a large number of cases of which I have had the first care. T. S., of Lee county Ala., a double talipes equino-varus from birth as shown in fig. 10, was, at the age of three, operated on by myself, assisted by Dr. W. T. Gautier of Columbus, Ga. After many fruitless attempts to use fixed dressings on him, I applied a dressing as seen in fig. 11, which may be used with a jointed sole shoe if desired; and is a most efficient way to employ it. This dressing, although not now my favorite one, was a success and an improvement on the fixed dressings. In twelve months I dismissed the patient as shown in fig. 12. He walks, runs and jumps like other children, and no one would suspect that this is his genuine photograph.

In my experience and observation the main difficulty in the

CASE VII.

*Fig.* 10. *Before any treatment.*

*Fig.* 11. *Elastic dressing.*

*Fig.* 12. *After* 12 *months use of elastic dressing.*

use of steel braces is that the foot must be almost exactly straight before they can be applied. If it is attempted to straighten the foot thus, it will cause such force and violence that the joint will be so swollen and painful that the patient will have the braces removed, and a failure is the consequence ; while on the other hand, if the foot is not sufficiently straight it will turn over in the brace in spite of all that can be done. Others have seen and contended with this trouble, for various attempts have been made to remove it. In the case of this young man, and in cases number two and three, they tried to do away with the trouble by reducing the varus with one brace and then the equinus by another. Certainly the results attained in these cases do not justify its imitation. Others have attempted to make the dressing conform to the deformed shape of the foot by placing a joint in the sole of the shoe, while others put an extra joint in the shaft of the brace. By these means it is attempted to remove the difficulty under consideration. I have often attempted to use the jointed sole shoe, but have always had to remove it. Dr. Sayre, its principal advocate, is led to its support by a course of mystified theorizing, by which he comes to the conclusion that the deformity exists only in the medio-tarsal junction. If the professor is correct in this conclusion the jointed sole is at least theoretically correct. But the conclusion is doubtful, which I hope I can show. It is argued, that as all the muscles of the leg except the gastrocnemius, soleus and plantaris are attached to the foot anterior to the medio-tarsal junction, any deformity produced by them must also be anterior to the ankle joint. From this he concluded that the deformity exists only in the medio-tarsal junction, leaving out of consideration the fact, that while these muscles are affected, producing varus, the muscles posterior to the ankle joint may also be affected, and produce deformity in the ankle joint, or equinus, as is often the case, the equinus being the most prominent and the last to be overcome. If there is deformity only in the medio-tarsal junction, why is contraction of the tendo-Achillis prominent, and why is its section necessary ? Then if equino-varus is a complex deformity, as its name indicates, partaking alike of the characteristics of both equinus and varus, it must partake of the only essential property of equinus, namely, deformity at the ankle joint. Then why place a ball and socket joint in the sole of the shoe? It certainly does not contribute to comfort. It is not necessary to the complete exercise of the foot, as there is no such joint in the foot. Nor is there any reason why all the force applied to the foot to reduce the deformity should be directed to one joint to the exclusion of the other. But this joint necessarily directs all the force to the medio-tarsal junction and should be abandoned.

Patients, after tenotomy, find it more comfortable to have this elastic dressing on than to be without it. No force or violence is necessary to its application. But it is applied gently while the foot is in almost its original shape. The straps are continually and efficiently yet mildly pulling the foot into position, at times when the patient is least conscious of it. He is not left for months to sit around before he may so much as attempt to put his foot to the ground, as they are when the brace is used as it was in Cases No. 1, No. 2 and No. 3. While by the elastic method, Case No. 2 walked with the dressing on in four days; No. 3 in ten days; No. 4 in four days; No. 6 in four days; No. 7 in thirty days. The time necessary for a cure is also quite different. With the elastic dressing many are cured in three or four months, and very few require more than twelve months treatment. While with the brace as we have seen many go ten, fifteen and twenty years. This young man has worn his sixteen and the end still is not in sight. This looks very much like wearing braces all a lifetime to be straight to die.

## Figures and Tables

**Fig. 1. f1:**
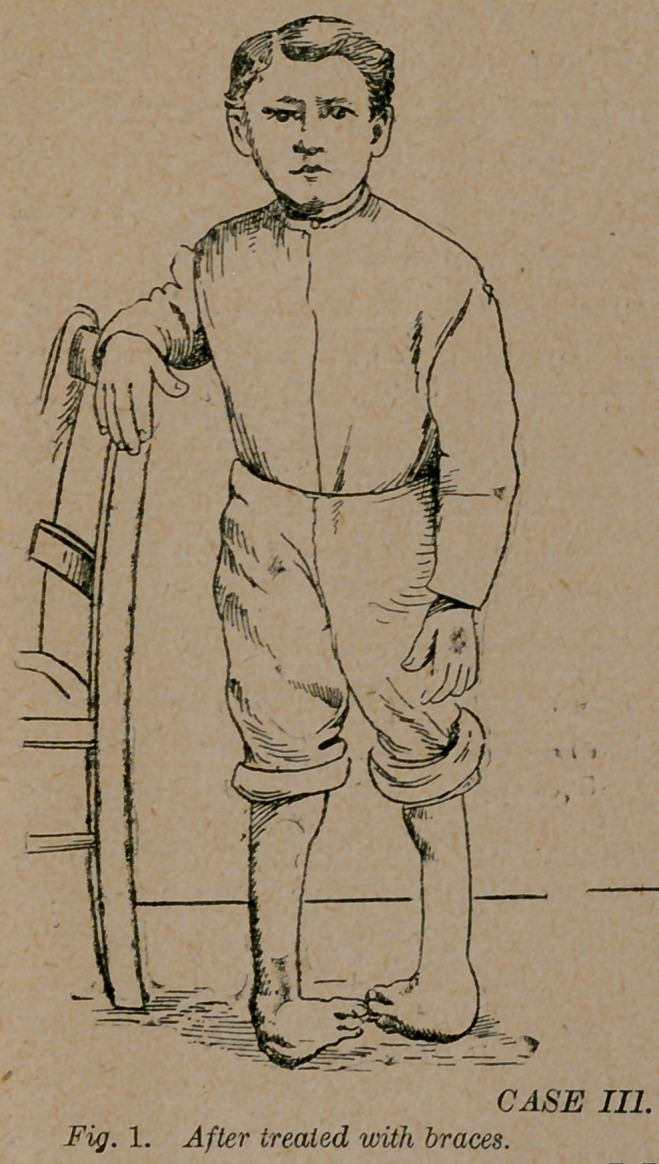


**Fig. 2. f2:**
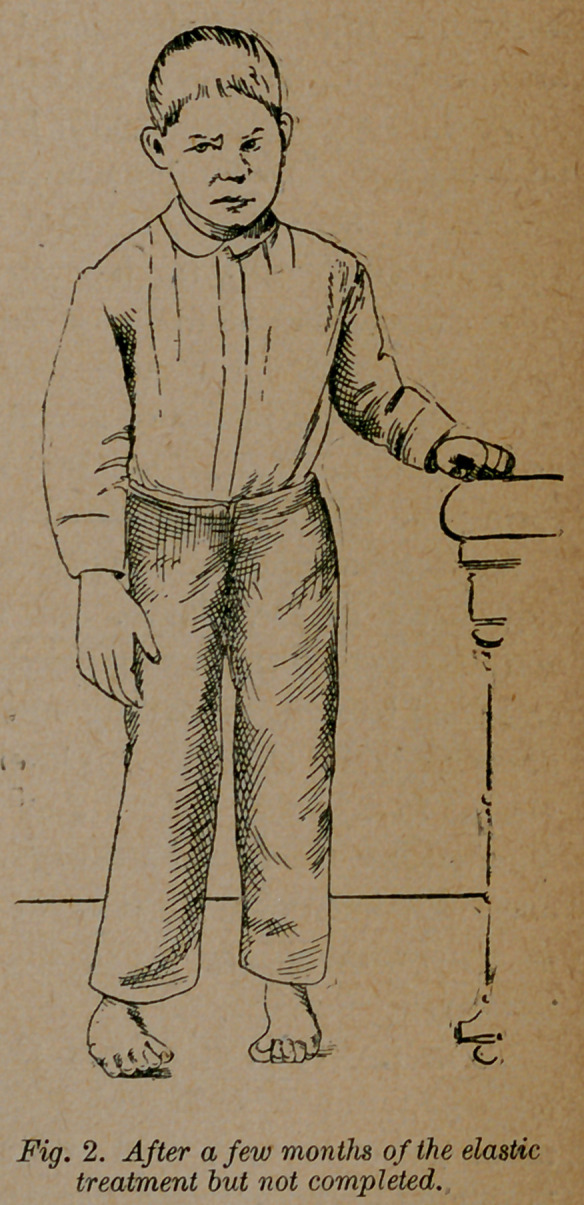


**Fig. 3. f3:**
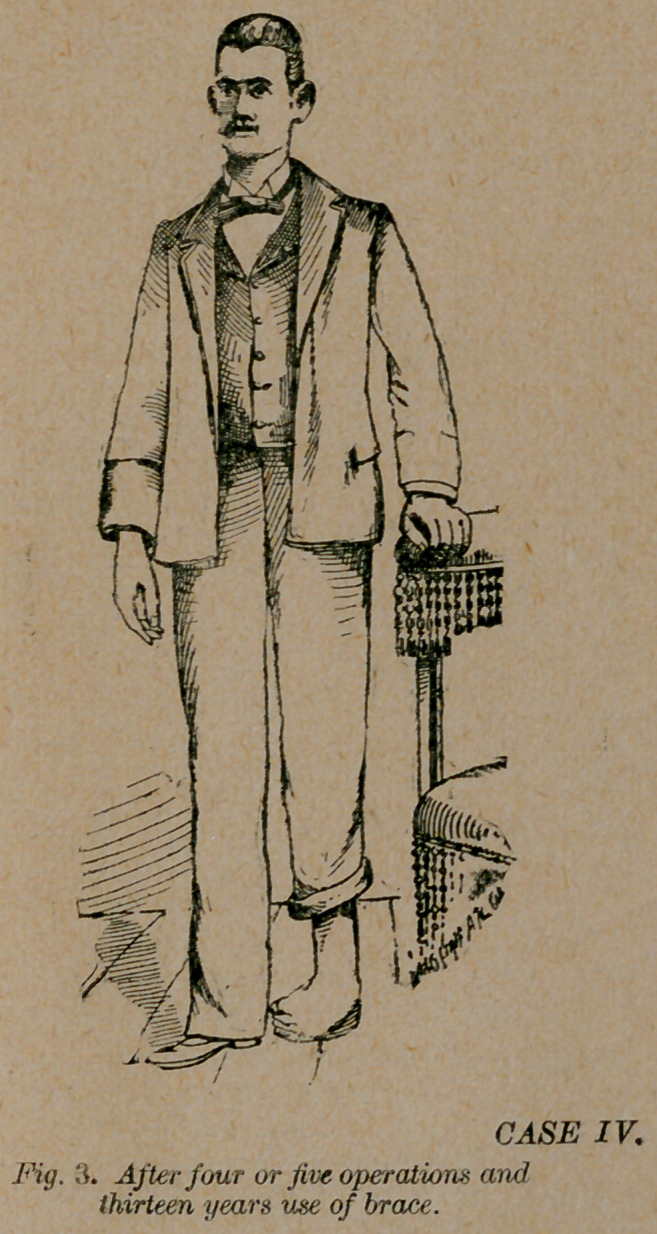


**Fig. 4. f4:**
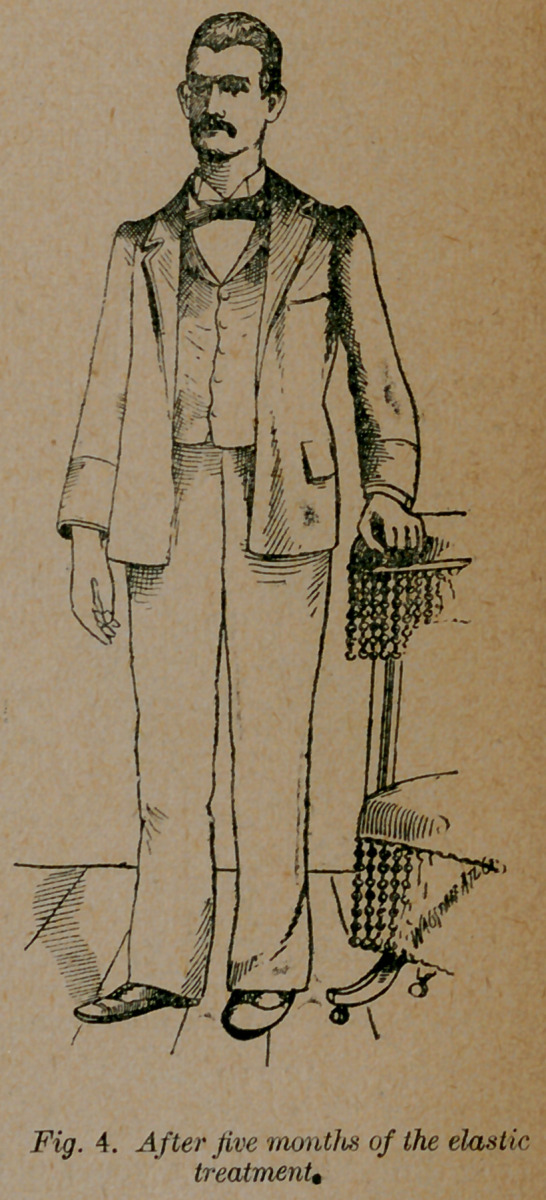


**Fig. 5. f5:**
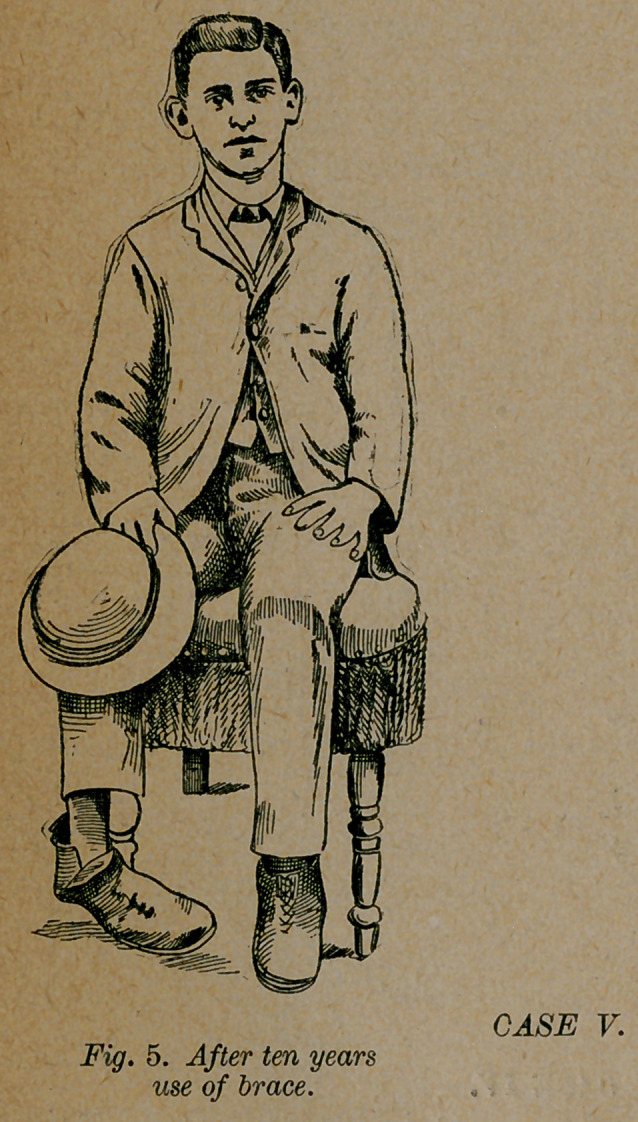


**Fig. 6. f6:**
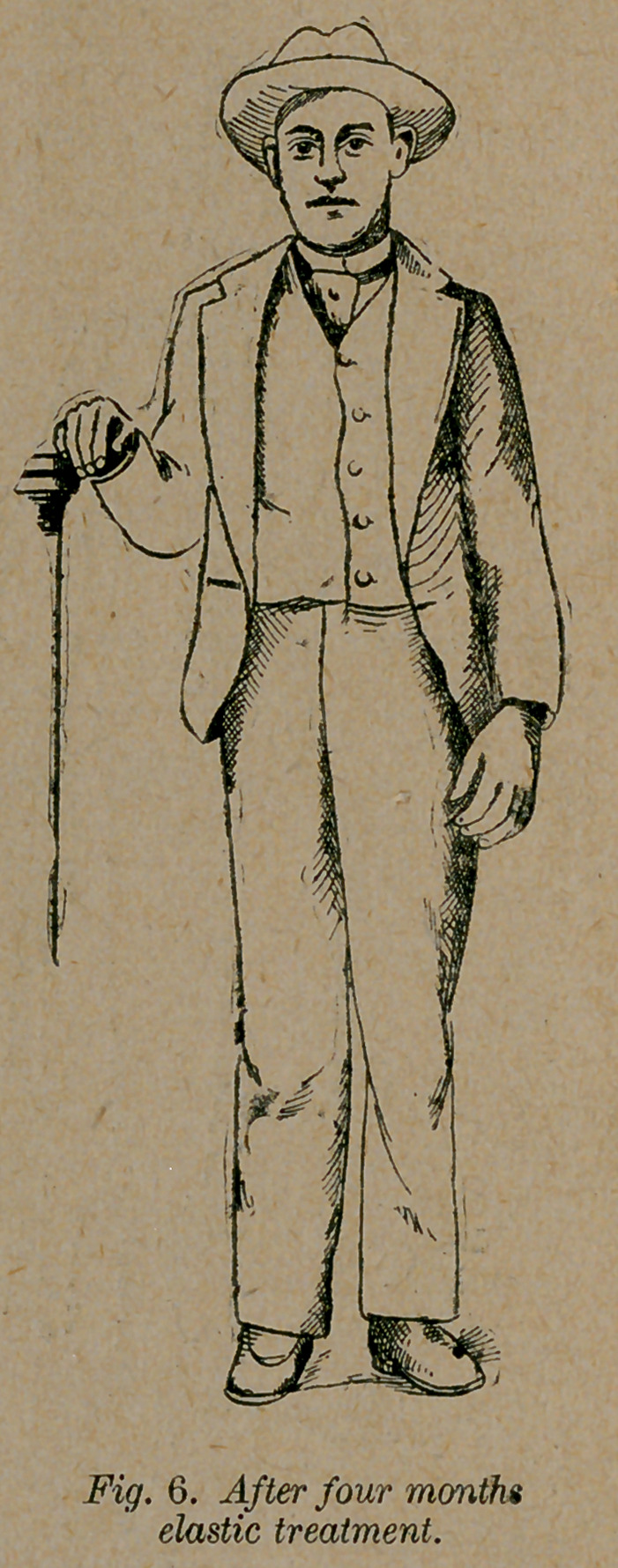


**Fig. 7. f7:**
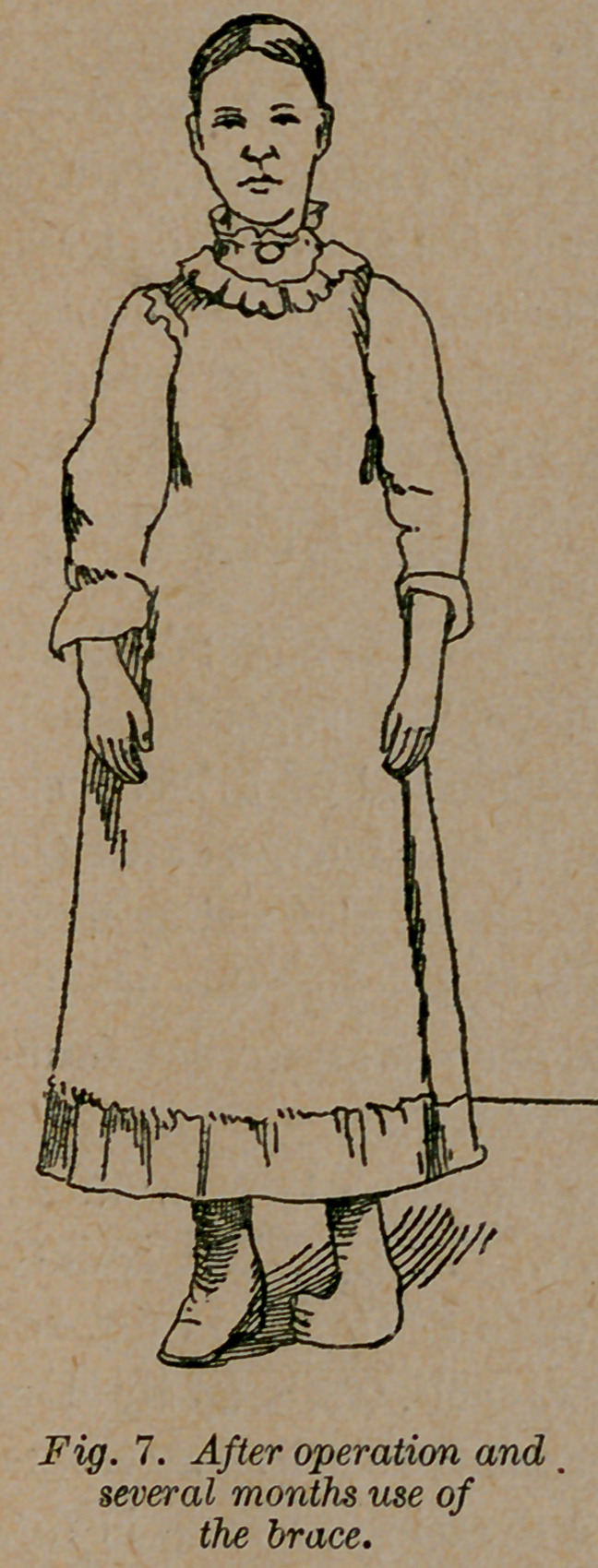


**Fig. 8. f8:**
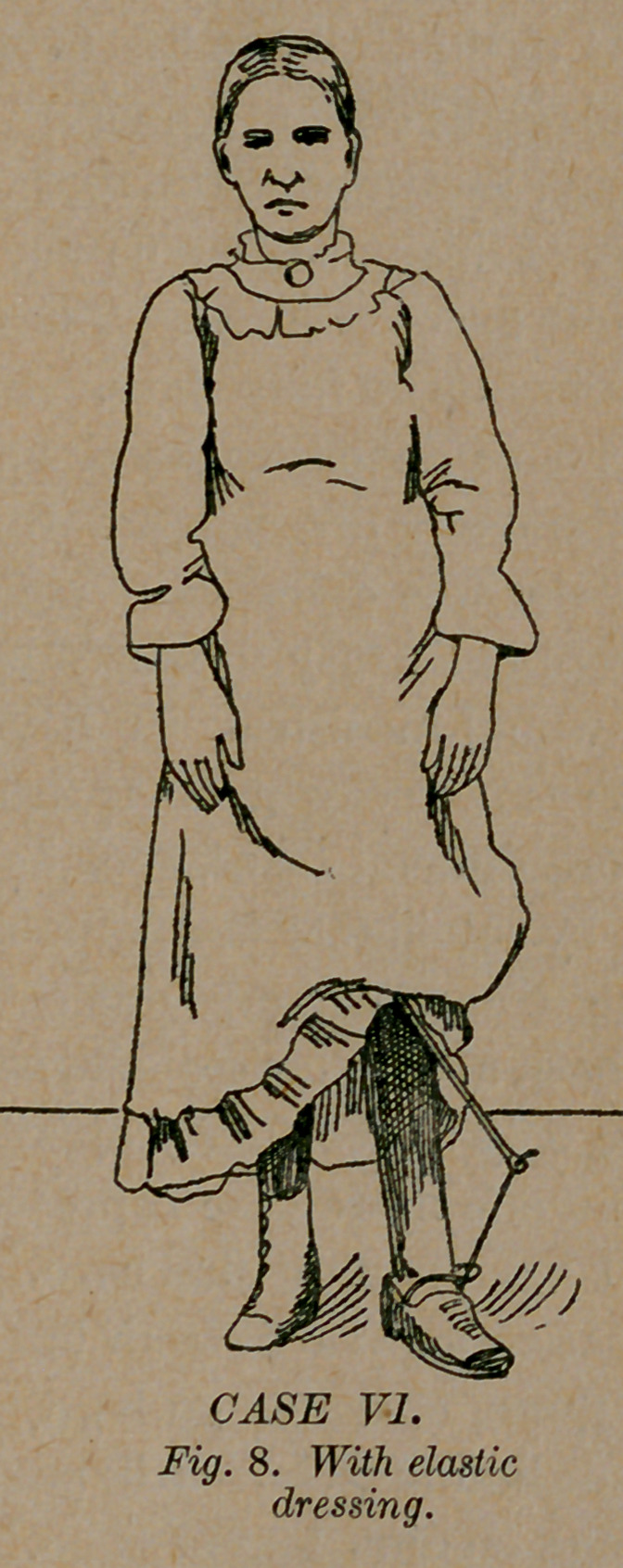


**Fig. 9. f9:**
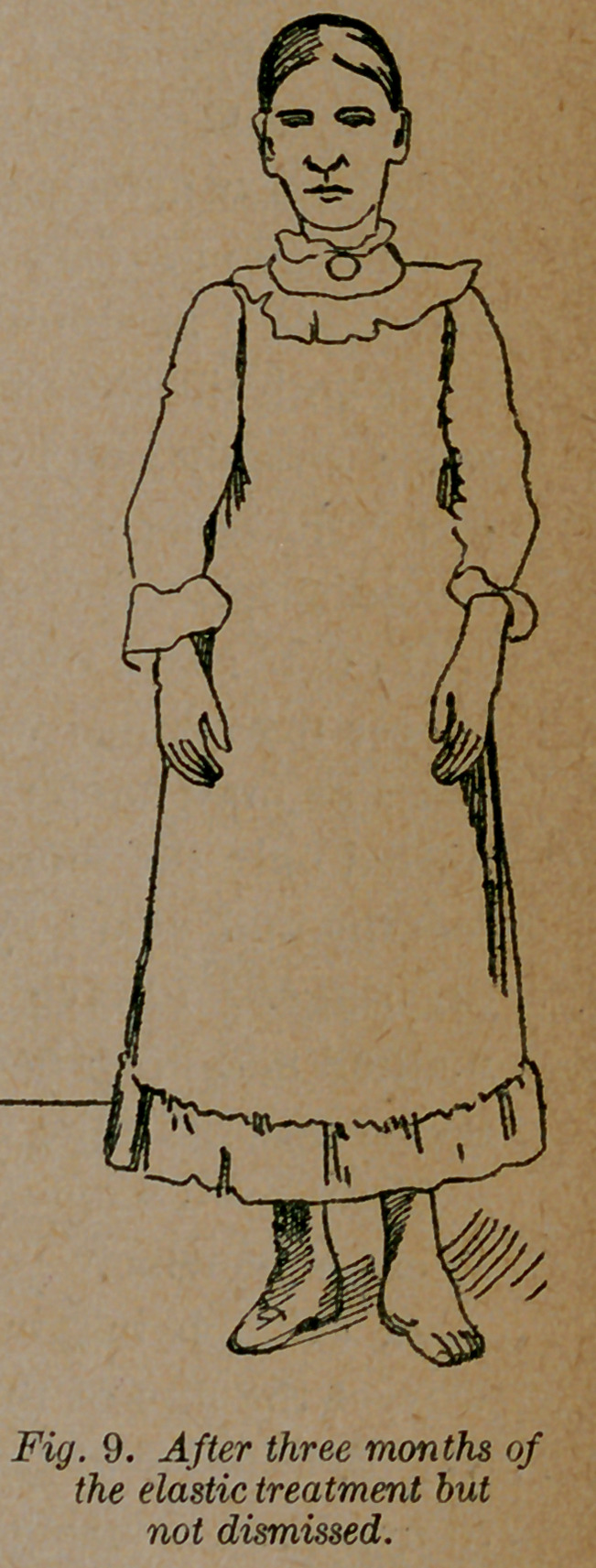


**Fig. 10. f10:**
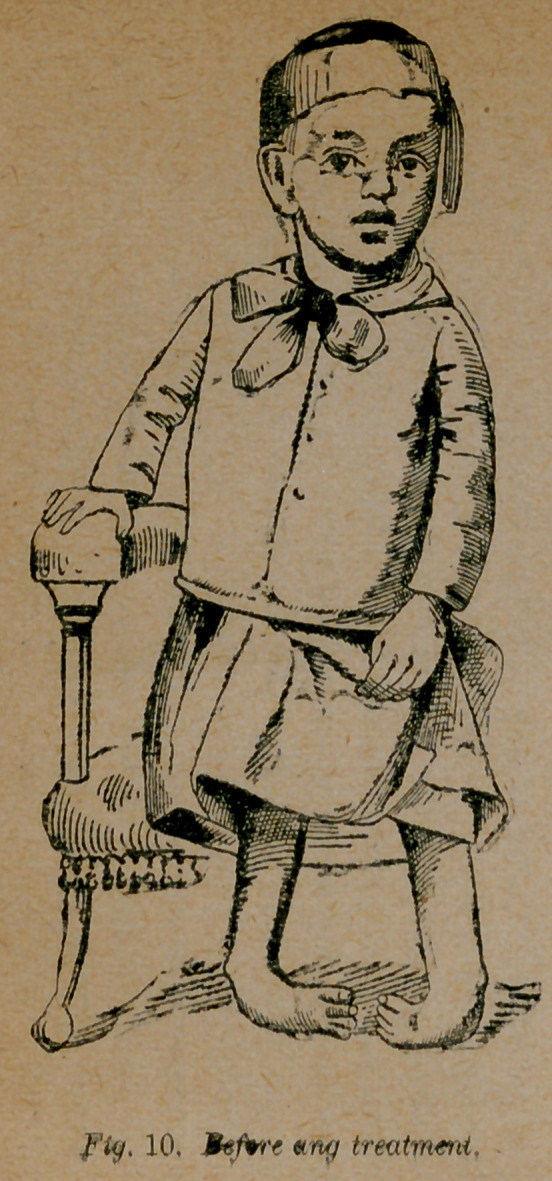


**Fig. 11. f11:**
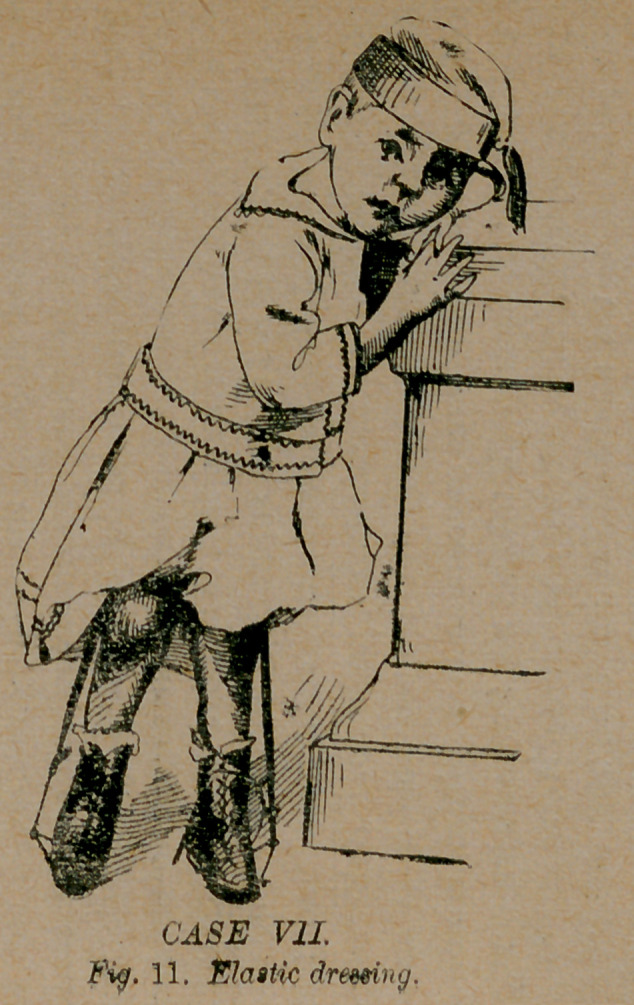


**Fig. 12. f12:**